# Supplementary material for: “Vulnerabilities and compound risks of escalating climate disasters in the Brazilian Amazon”
Source: Nat Commun. 2025 Nov 23;16:11579. doi: 10.1038/s41467-025-66603-0 (PMC12749845; doi:10.1038/s41467-025-66603-0)
Supplement: Supplementary file 1 — Description of Additional Supplementary Files [file 41467_2025_66603_MOESM1_ESM.pdf]

Description of Additional Supplementary Files

File name: Supplementary Data 1
Description: This Excel file contains the raw data and core analytical outputs that underpin Figures 1 to 5 in the main manuscript. The file includes multiple sheets, each corresponding to specific datasets and analyses. A detailed guide to the content, including variable definitions and data sources, is provided in the first sheet titled "Description".

File name: Supplementary Data 2
Description: This Excel file presents the step-by-step calculation of the Maximum Percentage of Population Affected per Municipality across the study period. These values are visualized in Figure 2B (map). All formulas and intermediate steps are shown to ensure transparency and reproducibility.
